# Poster Session I - A81 SMALL INTESTINAL BACTERIAL OVERGROWTH (SIBO) IN PATIENTS WITH NON-RESPONSIVE CELIAC DISEASE ATTENDING THE MCMASTER ADULT CELIAC CLINIC

**DOI:** 10.1093/jcag/gwaf042.081

**Published:** 2026-02-13

**Authors:** G Carr, A Verma, M Khaouli, J Blom, R Leong, A Liu, M Pinto-Sanchez

**Affiliations:** McMaster University, Hamilton, ON, Canada; McMaster University, Hamilton, ON, Canada; McMaster University, Hamilton, ON, Canada; McMaster University, Hamilton, ON, Canada; McMaster University, Hamilton, ON, Canada; McMaster University, Hamilton, ON, Canada; McMaster University, Hamilton, ON, Canada

## Abstract

**Background:**

Celiac disease (CeD) is an immune-mediated condition triggered by gluten consumption. Strict adherence to a gluten-free diet (GFD) remains the only effective treatment. Around 30% of CeD patients experience persistent symptoms despite attempting a strict GFD, a condition known as non-responsive celiac disease (NRCD). Among the various causes, small intestinal bacterial overgrowth (SIBO) is a recognized contributor to NRCD. However, its prevalence has not been evaluated in a Canadian CeD population.

**Aims:**

To determine the prevalence of SIBO and related clinical characteristics in a Canadian cohort of CeD patients.

**Methods:**

A chart review was conducted in CeD patients enrolled in the Adult Celiac Registry at a tertiary care center in Hamilton, Ontario. Patients with biopsy-proven CeD were included in the analysis. Demographic, diagnostic, and clinical data, including GFD adherence, symptoms, SIBO testing, and antibiotic response, were extracted. NRCD was defined as persistent gastrointestinal symptoms despite reported strict GFD adherence for at least 6 months. SIBO was diagnosed using hydrogen breath testing with a glucose substrate. A test was considered positive based on the North American Consensus, requiring either a rise in hydrogen of ≥ 20 parts per million (ppm) above baseline within 90 minutes, or a rise in methane of ≥ 10 ppm at any point during the test. Categorical data are reported as n (%) and continuous data as mean (SD).

**Results:**

We included 278 biopsy-proven CeD patients, and NRCD was identified in 104 (37.4%) of them. The mean age was 42.8 years, and 76.3% were female. Overall, 68.3% of patients were strictly adherent to the GFD. Among these, 16 (15.4%) had a positive test for SIBO and 10 of them reported symptom resolution after antibiotic therapy (4 unreported). The most common symptoms reported in patients with positive tests for SIBO were bloating in 12 (75%), abdominal pain in 8 (50%), and constipation in 8 (50%). There were no significant differences in frequency of symptoms between SIBO-positive and SIBO-negative NRCD patients.

**Conclusions:**

A subset of NRCD patients had a positive SIBO test and responded to antibiotic therapy, suggesting SIBO as a potentially treatable contributor to persistent symptoms in CeD. The similarity of symptoms between SIBO-positive and -negative patients underscores the need to consider other causes of NRCD in evaluation and management of these patients.

**Funding Agencies:**

McMaster University, Hamilton Health Sciences

